# Structured patient handoff on an internal medicine ward: A cluster randomized control trial

**DOI:** 10.1371/journal.pone.0195216

**Published:** 2018-04-19

**Authors:** Penny Tam, Aman P. Nijjar, Mark Fok, Chris Little, Alexandra Shingina, Jesse Bittman, Rashmi Raghavan, Nadia A. Khan

**Affiliations:** 1 Division of General Internal Medicine, Department of Medicine, University of British Columbia, Vancouver, British Columbia, Canada; 2 Division of Geriatric Medicine, Department of Medicine, University of British Columbia, Vancouver, British Columbia, Canada; 3 Department of Medicine, University of Toronto, Toronto, Ontario, Canada; 4 Division of Family Practice, Department of Medicine, University of British Columbia, Vancouver, British Columbia, Canada; 5 Center for Health Evaluation and Outcomes Sciences, University of British Columbia, Vancouver, British Columbia, Canada; Medical faculty RWTH Aachen, GERMANY

## Abstract

**Background:**

The effect of a multi-faceted handoff strategy in a high volume internal medicine inpatient setting on process and patient outcomes has not been clearly established. We set out to determine if a multi-faceted handoff intervention consisting of education, standardized handoff procedures, including fixed time and location for face-to-face handoff would result in improved rates of handoff compared with usual practice. We also evaluated resident satisfaction, health resource utilization and clinical outcomes.

**Methods:**

This was a cluster randomized controlled trial in a large academic tertiary care center with 18 inpatient internal medicine ward teams from January-April 2013. We randomized nine inpatient teams to an intervention where they received an education session standardizing who and how to handoff patients, with practice and feedback from facilitators. The control group of 9 teams continued usual non-standardized handoffs. The primary process outcome was the rate of patients handed over per 1000 patient nights. Other process outcomes included perceptions of inadequate handoff by overnight physicians, resource utilization overnight and hospital length of stay. Clinical outcomes included medical errors, frequency of patients requiring higher level of care overnight, and in-hospital mortality.

**Results:**

The intervention group demonstrated a significant increase in the rate of patients handed over to the overnight physician (62.90/1000 person-nights vs. 46.86/1000 person-nights, p = 0.002). There was no significant difference in other process outcomes except resource utilization was increased in the intervention group (26.35/1000 person-days vs. 17.57/1000 person-days, p-value = 0.01). There was no significant difference between groups in medical errors (4.8% vs. 4.1%), need for higher level of care or in hospital mortality. Limitations include a dependence of accurate record keeping by the overnight physician, the possibility of cross-contamination in the handoff process, analysis at the cluster level and an overall low number of clinical events.

**Conclusions:**

Implementation of a multi-faceted resident handoff intervention did not result in a significant improvement in patient safety although did improve number of patients handed off. Novel methods to improve handoff need to be explored.

**Trial registration:**

Registered at ClinicalTrials.gov: NCT01796756.

## Introduction

Efforts to improve patient safety and resident physician well-being resulted in an international push to limit resident work hours [1–2]. After the implementation of such restrictions in Canada, the United States, and Europe, the results are varied, with some studies showing worse patient outcomes, negative impacts on resident quality of life [1] with yet others showing a decrease in adverse events [3]. These changes emphasized the importance of “handoff”, a term used to describe the transfer of patient information from the outgoing physician of care to the incoming on-call physician [4].

At large institutions with high volumes of inpatients, handoff on all patients is not feasible, thereby requiring daytime physicians to prioritize a smaller subset of patients to convey potential issues on. From a survey of 26 physicians, it was noted that incomplete and omitted information in handoff was a significant issue and considered to lead to failure to anticipate and communicate problems to on-call physicians [5]. Previous work showed poor handoff specifically implicated in 20–24% of medical errors [4], while a more recent survey of Canadian residents showed that nearly half of respondents were directly involved in or witnessed an adverse event related to handoff [6]. Given this, the World Health Organization (WHO) and other regulatory bodies prioritized optimizing handoff as a top patient safety initiative [1,7–8].

A number of strategies for optimal handoff were suggested with standardization being noted most frequently [4]. Other suggested strategies include providing training or education and addressing environmental issues such as lighting, interruptions and noise [1,4,9]. Despite a call from the Accreditation Council for Graduate Medical Education (ACGME) to develop curricula [10], many residency programs do not educate trainees in the process of handoffs, with approximately 57% of Canadian residents learning this skill informally from other residents or staff [5]. Some of these strategies have recently been evaluated in a randomized trial of handoff methods in a surgical training program, demonstrating no clinically significant differences in patient outcomes [11]. We set out to evaluate implementation of standardized handoff among medical ward patients in a parallel controlled trial.

We conducted a cluster randomized controlled study to assess the effect of implementing a multi-faceted and standardized handoff intervention on the rates of handoff by residents. The intervention consisted of training and education on the type of patient to handoff, what information to handoff, and identifying a specific time and location for uninterrupted face-to-face handoff. We also assessed this intervention on resident satisfaction, and patient level outcomes including health resource utilization and clinical events. Clustering by ward teams was employed to minimize contamination of the structured handoff process between medical trainees within the same teams.

## Methods

### Study design

We conducted a cluster randomized-controlled trial (RCT) within the internal medicine inpatient wards at Vancouver General Hospital (VGH) between January 2013 and April 2013. Eligible ward medical teams consisted of one attending physician, 3–4 residents and 2–3 medical students. The physician staff and students are designated by a team color and change every four weeks. However, patients generally stay on their designated team throughout their hospital admission (96.6%). Ward teams that consisted of only one attending and one resident were not included in this study. All patients admitted to the eligible Internal Medicine wards were also included in this study.

Nine inpatient medical teams were randomized to intervention and nine teams were randomized to usual care by the PI (PT) by drawing lots as a manual method of simple randomization. There was no allocation concealment. The intervention was targeted at resident trainees within medical teams. Residents were placed on certain ward teams by the chief medical resident, who was not involved in this study. They were allocated, per usual methods, to ensure an equal number of senior and junior residents on each team. All residents on the intervention teams were included in the intervention, since any one of them could be providing handoffs. Patients admitted to various inpatient teams was in a generally random fashion, depending on bed availability.

### Study setting

VGH is a 950-bed academic, tertiary care center located in Vancouver, British Columbia. The internal-medicine service, also known as the Clinical Teaching Unit (CTU) has an average daily census of 100–110 medical patients. During daytime hours, the medicine service consists of six teams, each comprised of one attending physician and one to four residents and two to four medical students at various levels of training.

On-call clinical associates (CAs) are senior level residents (generally in post-graduate years 3–5) who cover evening shifts between 18:00–07:00 hours. The primary handoff period occurs from the daytime teams to a single CA who is responsible for all the CTU ward patients overnight.

### Intervention

The intervention group received a 45-minute training and education session on the importance and evidence for patient handoff, standardization of type of patient and information to handoff as well designating a fixed time (17:30–18:00) and dedicated quiet location for face-to-face handoff (4). One faculty member and one resident delivered the education session at the start of each monthly block to trainees. For the type of patient to handoff, trainees were instructed to handoff all patients who had any of: (i) investigations pending, (H) currently located in a high acuity unit, (A) abnormal vital signs in the preceding 24 hours, (N) newly admitted in the last 24 hours, and (D) dying. The hand off criteria (iHAND) was developed following a literature review and focus groups of eight clinical faculty members and six CAs. Validation of iHAND was done in a case control analysis of 90 inpatients, where the effectiveness of clinical gestalt, MEWS, and iHAND were evaluated using logistic regression analyses and receiver operating characteristic (ROC) curves [12]. The iHAND criteria showed a moderately significant correlation with requiring assessment overnight S1 File.

Trainees were also instructed to handoff information following the widely adopted SIGNOUT? mnemonic (Sick or do not resuscitate, Identifying data, General hospital course, New events of the day, overall health status, Upcoming possibilities with plan, Tasks to complete, ?Questions) [11]. Participants in the trainee session practiced handoffs to one another in small groups for the last 10–15 minutes with the facilitators supervising and providing feedback. Lastly, participants were given a pocket card with the mnemonics taught for use during handoff S3 File. The usual care group was given no specific instructions and handoff practices were left to the discretion of the team.

### Study outcomes

The primary outcome of the study was the rate of patient handoffs to the CA. Patient handoff events were only counted once per overnight shift per patient but patients may have had other handoff events during the course of their admission. In addition, total patient encounters represented an individual patient and included any and all documented handoffs, nursing questions, physical assessments, and actions taken by the CA overnight. The CAs completed data collection forms on all patients they received handoff on as well as patients that required assessment during the overnight shift S2 File.

We also measured the satisfaction of the handoff process as perceived by the CAs along with resident satisfaction. Health resource utilization was assessed using aggregate length of stay of patients admitted to the CTU and resource utilization for patients assessed by the CA overnight. Resource utilization, measured at the level of the patient, including radiographic imaging, blood tests, electrocardiogram, blood transfusions, IV fluid administration, and antibiotics were obtained through medical record review of all patients seen by the CA. Clinical endpoints analyzed at the level of the patient, included frequency of patients transferred to the Intensive Care Unit (ICU) and/or referred to the Critical Care Outreach Team (CCOT); aggregate rate of in hospital mortality of patients admitted to the CTU; and evaluation of medical errors of patients handed off to the CAs. Clinical endpoint data were collected through inpatient chart review and data on ICU transfers or CCOT referrals were provided by the ICU department. We only included ICU transfers and/or CCOT referrals occurring in the time frame of 18:00–07:00.

To further evaluate the effect of the intervention on patient safety, an additional outcome of medical errors was included in the study. A trained research assistant and physician team member (APN) blinded to the randomization of the patient conducted chart reviews on each patient handed off during the study period. For determination of medical errors, all examining physician notes, nursing notes, orders, and any incident reports were reviewed during the 48 hour period after a patient was handed off. The 48-hour limit was instituted to try and capture errors that might be directly attributed to handoff. All medical errors (including medication errors, procedure related errors, diagnosis or history and physical related errors, and falls) and preventable adverse events (any injury or harm to a patient arising from medical care delivery) were evaluated from chart review and were consistent with definitions from the IPASS study [3]. Chart review was possible on 94.3% of the original patient cohorts for evaluation of medication errors (446/473).

### Statistical analysis

We used intention to treat analysis. The sample size was powered to 80% to detect a 30% increase in proportion of patient handoff between the two groups with an alpha of 0.05 using the assumption that the average proportion of handoff is 5% (total sample size = 7560 patient nights). Assuming the average follow-up time is 10 days, the study will include 9 teams for the intervention and usual care group, respectively, and each team will take care of 45 patients.

For each outcome, the total number of events and total person-nights of each team were summarized. The event rate was calculated as per 1000 person-nights. Poisson regression model was conducted to compare the event rate between the two treatment groups. Team was the unit of analysis and total person-night was used as an offset in the Poisson regression. The relative risk of the two groups and its 95% confidence interval from Poisson regression model were reported. In the sensitivity analysis, regression model was performed with adjusting for the number of patients per resident in each team for resource utilization and clinical outcomes.

Comparison of proportion of medical errors was done using Chi-square test. Survey results of the CAs were compared using Bowker’s test of asymmetry between intervention and control groups, while trainee satisfaction survey results were examined using Chi-square test. Missing data from surveys were excluded from analysis. All analyses were conducted using SAS software, version 9.2 (SAS Institute). A p value <0.05 was considered statistically significant. This study was approved by the University of British Columbia and Vancouver Coastal Health Authority ethics boards. Informed consent of the residents was obtained by the voluntary participation in completing the surveys after reading the accompanying cover letter. This approach was approved by the Ethics Board. A waiver of consent was provided for patient level information since it was limited to chart review, involved minimal risk, and did not involve a therapeutic, clinical, or diagnostic intervention.

Registration in the clinicaltrials.gov registry was delayed till February 14, 2013, while recruitment for the study was from January 14, 2013 to April 7, 2013. This was inadvertent and due to a clerical error. However, the study was approved by the Ethics board May 3, 2012 and there were no changes to the protocol or primary end points from when recruitment began to the registration of the trial. The authors confirm that all ongoing and related trials for this intervention are registered.

## Results

**Fig 1:**
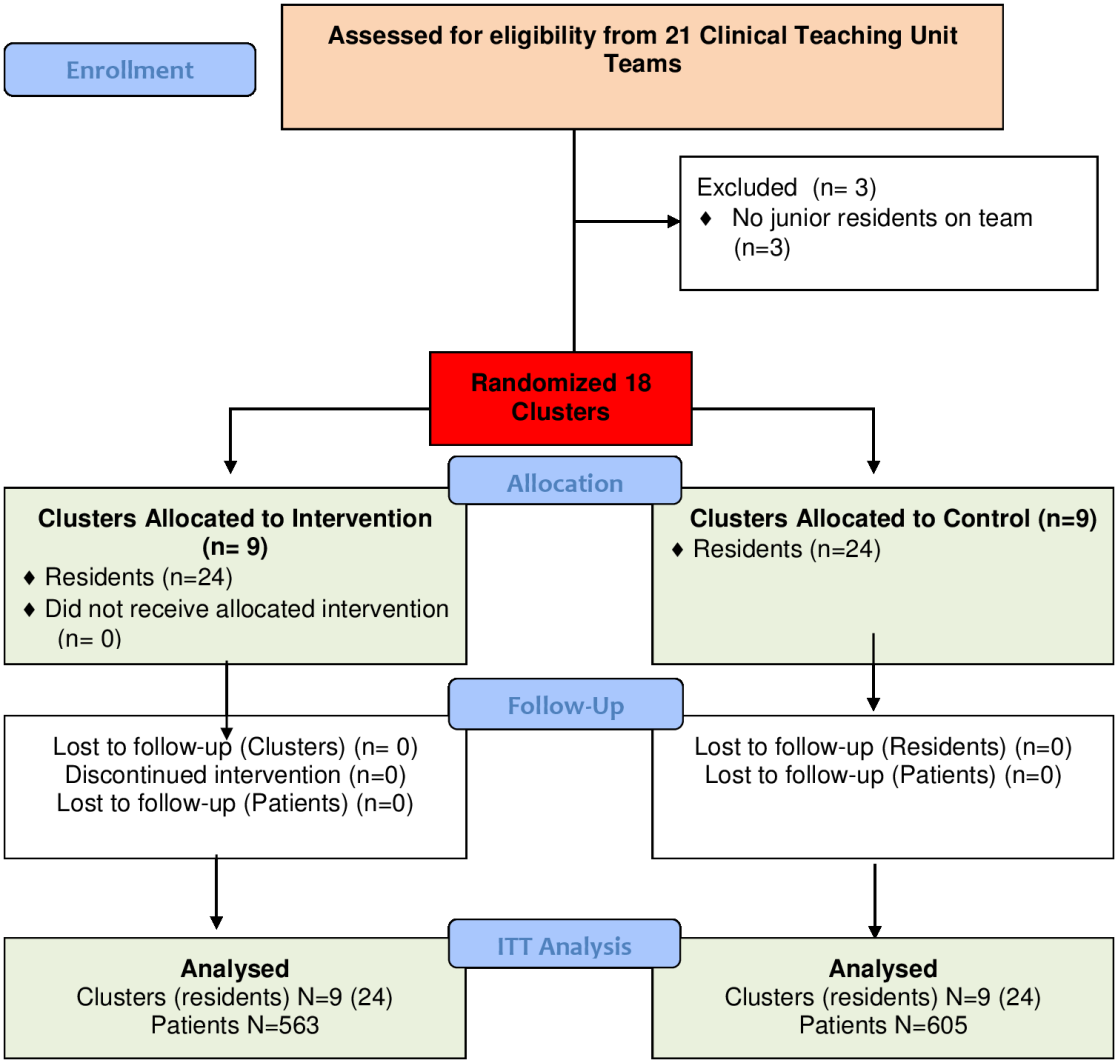
CONSORT flow diagram.

There were 48 residents and 1168 patients (8652 patient nights) during the study time (Fig 1). There was a similar number of residents and volumes of patients on the intervention and control teams (Table 1). Data on the total number of patients and their characteristics were provided by the hospital and were fairly balanced between groups. On the Clinical Teaching Units, most patients were elderly and the most common discharge diagnosis included pneumonia or influenza, sepsis, or gastrointestinal problems.

**Table 1:** Baseline characteristics of intervention and control groups.

Characteristics	Intervention	Control
**Clusters**	N = 9	N = 9
Number of Residents		
• Senior Residents	9	9
• Junior Residents	15	15
Number of Patients per Number of team residents mean (range)	8.7 (4.1–12.9)	9.6 (5.8–16.6)
Average Daily Patient team census	21.3	21.4
**Patients**	N = 563	N = 605
Mean age	66.7 (SD = 17.9)	65.5 (SD = 16.9)
Women (%)	264 (46.9)	280 (46.3)
Discharge Diagnosis (%)		
• Sepsis	9.9	8.1
• Pneumonia or Influenza	8.7	10.6
• Gastrointestinal	8.4	9.9
• Other	73.0	71.4

Abbreviations

SD: Standard deviation

Over the study period, the CA’s recorded a total of 1,150 patient encounters over the study duration (8652 person-nights). There were 599 patient encounters recorded in the intervention group and 551 in the control group. Of the 1,150 there were 265 patient handoffs (patients formally handed off to the CA) in the intervention group and 208 in the control group. In the intervention group, 49% (130/265) of the handoffs occurred face-to-face and 41.5% occurred over the phone. For the control group, face-to-face handoff occurred 38.5% (80/208) of the time, while 51.9% was over the phone.

### Process outcomes

The primary outcome of the rate of patient handoffs over to the CA by the primary team at the start of the overnight shift is demonstrated in Table 2. The handoff intervention group demonstrated a significantly higher proportion of patient handoffs to the CA (62.90/1000 person-days vs. 46.86/1000 person-days, p-value = 0.002) compared with usual practices.

**Table 2:** Process, healthcare resource utilization and patient clinical outcomes.

Outcomes	Intervention	Control	RR [95%CI]	p-value*
	Per 1000 person days (no. of events)			
**Process Outcomes**				
Proportion of patients handed off to CA	62.90 (265)	46.86 (208)	1.34 [1.12,1.61]	**0.002**
Proportion of patients handed off to CA	0.47 (2)	0.68 (3)	0.70 [0.12,4.20]	0.70
**Health Resource Utilization**				
Proportion of patients receiving resource utilization overnight	26.35 (111)	17.57 (78)	1.50 [1.12,2.00]	**0.01**
• IV fluids	8.78 (37)	7.66 (34)	1.15 [0.72,1.83]	0.56
• Antibiotics	2.85 (12)	1.13 (5)	2.53 [0.89,7.18]	0.08
• Blood products	3.09 (13)	1.13 (5)	2.74 [0.98,7.68]	0.06
• CT	0.71 (3)	0.23 (1)	3.16 [0.33,30.39]	0.32
• X-ray	4.27 (18)	3.38 (15)	1.26 [0.64,2.51]	0.50
• Ultrasound	0.47 (2)	0 (0)	NA	NA
• ECG	6.41 (27)	3.38 (15)	1.90 [1.01,3.57]	**0.047**
• Blood tests	14.24 (60)	11.94 (53)	1.19 [0.82,1.73]	0.35
**Patient Clinical Outcomes**				
Proportion of patients transferred to ICU	1.90 (8)	1.58 (7)	1.20 [0.44,3.32]	0.72
Proportion of patients evaluated by CCOT	6.65 (28)	7.43 (33)	0.89 [0.54,1.48]	0.66
In-hospital mortality rate	10.92 (46)	11.71 (52)	0.93 [0.63,1.39]	0.73
**Patients Handed Off**	**(N = 250)**	**N = (196)**		
All Medical Errors (%)	12 (4.8)	8 (4.1)	1.18 [0.49,2.82]	0.72
• Preventable Adverse Events	2 (0.8)	3 (1.5)	0.52 [0.09,3.10]	0.47

Abbreviations

CA: Clinical Associate

ICU: Intensive Care Unit

CCOT: Critical Care Outreach Team

*P-value from Poisson regression

Other outcomes included resident and CA satisfaction. Resident participation in the survey was low with a 62.5% response rate (30/48). There was no difference in the proportion of patients where the CA did not receive necessary handoff information. Satisfaction with the handoff process from both the CA and CTU resident perspective was not significantly different between intervention and control groups as shown in Table 3.

**Table 3:** Handoff satisfaction of clinical associates and trainees, comparison between intervention and control.

**CA Questionnaire (N = 23)**	**Intervention (%) Strongly Agree or Agree**	**Control (%) Strongly Agree or Agree**	**p-value**
I am satisfied with the daily handoff process	73.9	65.2	0.80
I receive sufficient handoff for my CA shift	65.2	60.9	0.81
I receive handover for MOST patients that in my opinion should have been handed off	65.2	56.5	0.68
I am satisfied with time spent on handoff	87.0	73.9	0.89
*p-value from Bowker’s test of symmetry			
**CTU Resident Questionnaire**	**Intervention (N = 17) Percent Strongly Agree or Agree**	**Control (N = 13) Percent Strongly Agree or Agree**	**p-value**
I am satisfied with the daily handoff process	62.5	78.9	0.32
When I handoff, I am confident that proper follow-up will occur.	79.2	84.2	1.00
I am satisfied with the amount of time spent handing off my patients to the CA	58.3	73.7	1.00
I am satisfied with the care my patients received by CA overnight.	83.3	84.2	0.77
*p-value from Chi-square test			

### Resource utilization

Average hospital length of stay was not significantly different either (9.25 vs. 9.28 days, p-value = 0.97). However, the proportion of patients receiving additional resource utilization was increased in the intervention group compared with the control group. Individual health resource utilization components were not significantly different between groups (Table 2).

### Clinical outcomes

Table 2 also includes secondary clinical outcomes of ICU transfer, CCOT referral, and in-hospital mortality, none of which were significantly different. Medical errors and potentially adverse events were infrequent and not significantly different between the two arms (Table 2).

### Sensitivity analysis

We adjusted for clustering in resource utilization and clinical outcomes, and the findings were generally unchanged except that use of blood products was significantly associated with the intervention (adjusted RR 2.94, 95%CI: 1.03–8.43, p = 0.04; unadjusted RR 2.74, 95%CI:0.98–7.68, p = 0.06).

## Discussion

The implementation of a multi-faceted structured handoff program resulted in significantly more patients being handed off to the overnight physician and increased resource utilization compared with non-standardized handoff practices, but no significant differences in clinical outcomes or medical errors.

The bundled intervention demonstrated increased patient handoff and health resource utilization compared with usual handoff practices. These findings are consistent with other studies [13–15] and recommendations by the ACGME to improve handoff processes [16]. Our intervention may have led to increased health resource utilization because the CA does often receive specific instructions for investigations to be followed up overnight. Increased use of ECGs may reflect a common request to evaluate a complaint of chest pain. Increased use of blood products may reflect instructions to transfuse blood given a specific hemoglobin result. These findings suggest that a structured training program on handoff with feedback can lead to improved handoff practices. From the resident and CA surveys, satisfaction with handoff was modest and not different in both intervention and control group. However, response rates were low, limiting interpretation of these findings. Resident satisfaction with formalized handoffs has already been identified in a number of studies [14–15,17].

The research demonstrating a significant impact of formal and standardized handoff processes in terms of resident training and clinical outcomes is limited. Our negative results on patient outcomes are consistent with other studies [18–20]. Using a case-based computer simulation to provide opportunities to teach and practice handoffs, a study by Johnson et al. on pediatrics residents did not find any significant differences in patient outcomes such as rapid response calls or transfers to the ICU with implementation of the module [18]. Formalized handoff processes can result in increased confidence among medical providers and a perceived decrease in “near-miss” events [19], but no demonstration of an actual improvement in patient outcomes. Use of face-to-face handoff also did not show improved patient outcomes among hospitalized medical patients [20]. In a prospective pre-post test study by Starmer et al., handoff on all patients using a multifaceted handoff bundled intervention in 9 pediatric inpatients centers showed a 23% reduction in medical error rates with handoff practices [21]. However, this study design used a time series analysis that is at risk for bias for differing co-interventions at different time points and temporal changes in outcomes. Outcomes were assessed on all patients, not just those seen by on-call physicians. Further, this study’s intervention of handing off all in-patients is not easily applied to high patient volume settings. There may be other efforts required to show a handoff bundle is effective in reducing medical errors or improving patient outcomes. The strongest and most recent randomized trial of formalized patient handoff in a surgical residency program did not show any difference in mortality, medical errors or length of stay [11]. This may indicate a need for more tailored handoff strategies for different settings. Others have commented on the need for robust information systems that involve an effective electronic medical record along with a sustainable cultural change program [22].

The potential reasons for the negative findings in change in clinical outcomes between our two groups are likely multi-fold. Despite an increase in patients handed off, we did not identify any significant differences in patient outcomes. Handoff to the evening CA traditionally occurs for the seriously unwell patients on the ward as standard of care and thus the residents assigned to the control group may have also similarly identified and handed off patients at high risk for being seen by overnight physicians thus minimizing differences in ICU and CCOT transfers. Other patient clinical deterioration events may also be less or difficult to predict so handoff on a select subset of patients may miss a significant proportion of patients needing evaluation by the overnight physician. Furthermore, overnight CAs are, in general, experienced senior-level residents. One can postulate that whether they received a patient handoff or not, CA’s were able to stabilize and manage patients quickly and appropriately by reviewing the chart once notified by nurses. Also, our medical error rate was lower than expected. Safety measures built into our system such as physician alerts for abnormal laboratory values, modified assignment nursing staff allowing for closer monitoring of sicker patients and residents on-call in the emergency department overnight may have allowed for closer follow-up on patient issues identified during the daytime.

The strengths of the study include a parallel controlled trial design and multiple outcomes measured in a high volume patient setting. We also used a multi-faceted intervention that included not only mnemonics, but also observation and feedback during the education session and a specific time and location for uninterrupted face-to-face handoff. Our study focused on the internal medicine wards with a general census of over 100 patients, common in many general internal medicine residency programs. However, our study has limitations. Data were dependent on the accurate record keeping by the CA, however there is no evidence to suggest a difference in accuracy between the intervention and control arm. Although we employed clustering in our intervention, there may have been some contamination in the handoff process between intervention and control. Conducting the analysis at the cluster level did not enable us to take advantage of patient level analysis, such as variation in cluster size and allowing adjustment for patient level characteristics. The number of clinical events including serious medical errors was low in our study, thus limiting our ability to detect smaller changes in these events. Results from our survey were limited due to lower than expected participation in the survey. Our study was carried out in a high volume academic tertiary care center with a single experienced resident covering overnight shifts and as such, our findings may not be generalized to lower volume hospitals or hospitals without trainees. Finally, we did not explore the effect of having a single CA covering over 100 patients and whether having more physicians to share the workload along with improved handoff would improve patient outcomes.

## Conclusions

While there is widespread recognition for the need in creating a handoff process in the inpatient hospital setting [23], an evidence-based approach for internal medicine residency programs remains to be determined. Our handoff bundle of resident training and education, standardization, mnemonics, and face-to-face handoff did not succeed in an improvement in general patient outcomes in a high volume in patient medical service. Novel methods to improve clinical outcomes associated with handoff need to be explored.

## Supporting information

S1 File IHAND derivation. (DOCX)

S2 File CA data collection sheet. (DOCX)

S3 File Education session pocket card. (DOCX)

S4 File CONSORT checklist. (DOCX)

S5 File Trial protocol. (DOCX)

S6 File Handoff data file. (XLSX)
