# Supplementary material for: Structured patient handoff on an internal medicine ward: A cluster randomized control trial
Source: PLoS One. 2018 Apr 19;13(4):e0195216. doi: 10.1371/journal.pone.0195216 (PMC5908079; doi:10.1371/journal.pone.0195216)
Supplement: S1 File — (DOCX) [file pone.0195216.s001.docx]

**Appendix A**

*Who should be handed-over*

This case control study included 90 in-patients, comprising 32 patients requiring overnight assessment (cases) and 58 patients not requiring overnight assessment (controls). Models based on demographic, physiologic variables, and resource utilization prior to handover were used to predict which patients would be seen by the CA. The effectiveness of gestalt, MEWS, and iHAND were evaluated using logistic regression analyses and receiver operating characteristic (ROC) curves.

Table A: Population Characteristics

| Characteristics | Required overnight assessment (N=32) | Did not require overnight assessment (N=58) |
| --- | --- | --- |
| Population size (%) | 35.6 | 64.4 |
| Age, mean±SD years | 65.7 (18.0) | 62.5 (19.2) |
| Male sex, % | 59.4 | 55.2 |
| Admitting diagnoses (%) |  |  |
| Congestive heart failure | 15.6 | 3.4 |
| Sepsis | 15.6 | 17.2 |
| GI bleed | 12.5 | 3.4 |
| Liver disease | 12.5 | 6.9 |
| Pneumonia | 3.1 | 12.1 |
| Delirium | 3.1 | 5.2 |
| Other | 37.5 | 51.7 |
| Average maximum heart rate (beat per minute) | 95.7 | 89.8 |
| Average maximum systolic blood pressure (mmHg) | 130.1 | 135.4 |
| Average minimum diastolic blood pressure (mmHg) | 57.8 | 63.3 |
| Average maximum respiratory rate (breaths per minute) | 20.9 | 19.9 |
| Average maximum temperature (°C) | 37.1 | 37 |
| Average minimum Oxygen saturation (%) | 95.4 | 96.8 |
| Level of consciousness (%) |  |  |
| Alert | 87.5 | 91.4 |
| Responding to voice | 9.4 | 5.2 |
| Responding to pain | 3.1 | 0.0 |
| Unresponsive | 0 | 1.7 |
|  |  |  |
| Newly abnormal laboratory value (%) | 62.5 | 48.3 |
| Investigation ordered during day and resulted at night (%) | 53.1 | 27.6 |
| Imaging ordered in last 24hrs, (%) | 53.1 | 34.5 |
| Admit less than 24hrs (%) | 31.3 | 13.8 |
| Code status (%) |  |  |
| Full code | 50.0 | 79.3 |
| DNR | 43.8 | 19.0 |
| Comfort care | 6.3 | 1.72 |
| Location (%) |  |  |
| Standard bed | 87.5 | 93.1 |
| High acuity unit | 6.3 | 5.2 |
| Emergency ward | 6.3 | 1.72 |

Of the patients seen overnight, 34% had been handed-off by the daytime medical team and of the patients not seen overnight, 26% had been handed-off (Table 14). Having been handed-off was not associated with requiring overnight assessment (Odds ratio (OR) 1.50, 95% confidence intervals (CI) 0.59-3.83).

The MEWS score is not significantly associated with requiring assessment overnight (OR 0.96, 95% CI 0.75-1.24), while the iHAND score was significantly correlated (OR 1.93, 95% CI 1.24-3.02). A MEWS cut point of three has moderate specificity (75.9%) and poor sensitivity (46.9%) for needing to be assessed overnight. An iHAND score of less than one has good sensitivity (87.5%) for not requiring assessment overnight, while an iHAND of 2 or higher has moderate specificity (77.6%) for requiring assessment overnight. Sensitivity and specificity of other cut points are shown in Table 14.

Table A.2: Sensitivity and specificity of handoff, MEWS and iHAND in predicting those assessed overnight

|  |  | Assessed overnight | | Sensitivity, % (95% CI) | Specificity, % (95% CI) |
| --- | --- | --- | --- | --- | --- |
|  |  | Yes | No |  |  |
| Handed-off | Yes | 11 | 15 | 34.4 (19.2-53.2) | 74.1 (60.7-84.4) |
|  | No | 21 | 43 |  |  |
| MEWS ≥ 2 | Yes | 21 | 29 | 65.6 (46.8-80.8) | 50 (36.7-63.3) |
|  | No | 11 | 29 |  |  |
| MEWS ≥ 3 | Yes | 15 | 14 | 46.9 (29.5-65.0) | 75.9 (62.5-85.7) |
|  | No | 17 | 44 |  |  |
| MEWS ≥ 4 | Yes | 9 | 9 | 28.1 (14.4-47.0) | 84.5 (72.1-92.2) |
|  | No | 23 | 49 |  |  |
| iHAND ≥ 1 | Yes | 28 | 35 | 87.5 (70.1-95.9) | 39.7 (27.3-53.4) |
|  | No | 4 | 23 |  |  |
| iHAND ≥ 2 | Yes | 16 | 13 | 50.0 (32.3-67.8) | 77.6 (64.4-87.1) |
|  | No | 16 | 45 |  |  |
| iHAND ≥ 3 | Yes | 11 | 6 | 34.4 (19.2-53.2) | 89.7 (78.2-95.7) |
|  | No | 21 | 52 |  |  |

Patients that were not seen tended to have lower MEWS and iHAND scores (Figure 1). The MEWS score is not significantly associated with requiring assessment overnight (OR 0.96, 95% CI 0.75-1.24), while the iHAND score was significantly correlated (OR 1.93, 95% CI 1.24-3.02). A MEWS cut point of three has moderate specificity (75.9%) and poor sensitivity (46.9%) for needing to be assessed overnight.


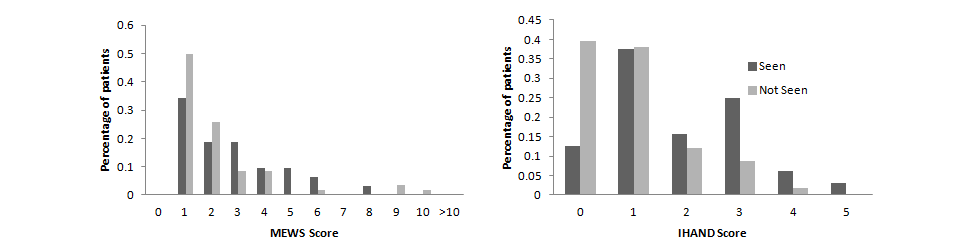


Figure A: Distribution of MEWS and iHAND scores for patients who required assessment overnight (n=32) and those who did not require assessment overnight (n=58)

The AUC of the MEWS score (0.613) indicates MEWS does not significantly discriminate between those requiring or not requiring assessment overnight. The AUC for iHAND indicates iHAND does significantly discriminate between those seen overnight and those not seen. The result of bootstrapping internal validation with 600 repetitions shows the bias corrected AUC is 0.702.


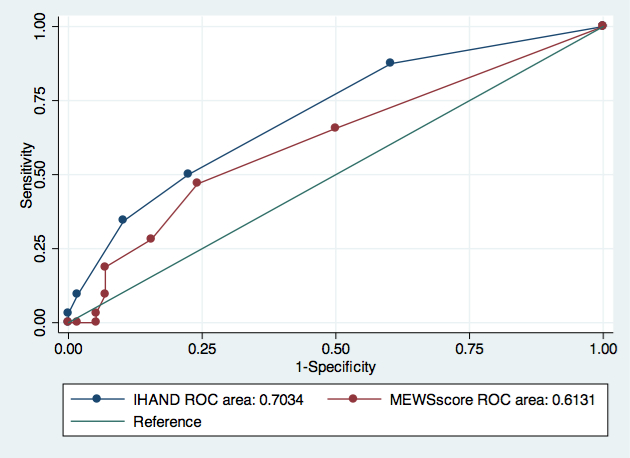


Figure A.2: Receiver operator characteristic curve for the iHAND and MEWS score.
