# Supplementary material for: Structured patient handoff on an internal medicine ward: A cluster randomized control trial
Source: PLoS One. 2018 Apr 19;13(4):e0195216. doi: 10.1371/journal.pone.0195216 (PMC5908079; doi:10.1371/journal.pone.0195216)
Supplement: S2 File — (DOCX) [file pone.0195216.s002.docx]

**CTU RED**

| Patient name:  Received handover Y☐ N☐  Face-to-face ☐  Phone ☐  Text ☐ | Notes: | Concerns about handover received for this patient?   - Yes. Comments: | Handback to CTU for am: |
| --- | --- | --- | --- |
| Patient name:  Received handover Y☐ N☐  Face-to-face ☐  Phone ☐  Text ☐ | Notes: | Concerns about handover received for this patient?   - Yes. Comments: | Handback to CTU for am: |
| Patient name:  Received handover Y☐ N☐  Face-to-face ☐  Phone ☐  Text ☐ | Notes: | Concerns about handover received for this patient?   - Yes. Comments: | Handback to CTU for am: |
| Patient name:  Received handover Y☐ N☐  Face-to-face ☐  Phone ☐  Text ☐ | Notes: | Concerns about handover received for this patient?   - Yes. Comments: | Handback to CTU for am: |
