# Supplementary figures and images for: Structured patient handoff on an internal medicine ward: A cluster randomized control trial
Source: PLoS One. 2018 Apr 19;13(4):e0195216. doi: 10.1371/journal.pone.0195216 (PMC5908079; doi:10.1371/journal.pone.0195216)

Education Session Pocket Card


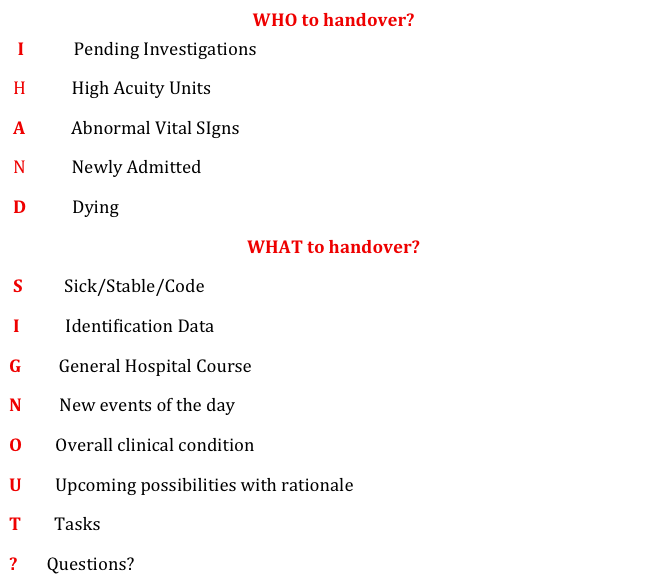

Supplement: S3 File — (DOCX) [file pone.0195216.s003.docx]
