# Supplementary material for: Structured patient handoff on an internal medicine ward: A cluster randomized control trial
Source: PLoS One. 2018 Apr 19;13(4):e0195216. doi: 10.1371/journal.pone.0195216 (PMC5908079; doi:10.1371/journal.pone.0195216)
Supplement: S5 File — (DOCX) [file pone.0195216.s005.docx]

**CMPA Grant Program 2011 - Proposal for Risk management program development**

**TITLE, PRINCIPLE INVESTIGATOR (PI) AND CONTACTS**

Quality Improvement in Handover of General Internal Medicine In-patients

Principal Investigator - Dr. Penny Tam

CMPA member number 20062222

Clinical Instructor, Department of Medicine, Division of General Internal Medicine, University of British Columbia

BSc, MD, FRCPC

University of British Columbia Department of Medicine

Gordon and Leslie Diamond Health Care Centre

7th Floor – General Internal Medicine Clinic

2775 Laurel Street

Vancouver, BC V5Z 1M9

Email: pennytam@interchange.ubc.ca

**PROJECT DESCRIPTION**

Studies have identified inadequate handover as a cause of adverse events, inefficient work and malpractice cases. It is widely recognized that miscommunication during patient handover can jeopardize patient safety and is preventable using a number of strategies identified in the literature. The PI of this proposed project recently completed a pilot Quality Improvement (QI) project at a major teaching hospital in Toronto evaluating the outcomes of a multi-faceted handover program on both residents and patients. The study was underpowered to show a significant change after implementation of the program due to the short study period, but the conclusion was that longer and larger scale studies are required.

At one of the major teaching hospitals in Vancouver, British Columbia there is no formal handover process for the General Internal Medicine in-patient service where multiple handovers occur. The goal of this project is to implement a multi-faceted handover program to address this important patient safety issue. The program will include education and training, face-to-face handover and standardization of verbal and written handover, all derived from the literature and lessons learned from the pilot project completed in Toronto. This program will contribute to the existing literature by evaluating the impact of handover strategies and whether changes in resident satisfaction and more importantly, patient outcomes can be measured and demonstrated after implementation of a handover program.

**BACKGROUND**

Patient handover has become the focus of many organizations worldwide and many studies have identified deficiencies in handover (1). In one study, a third of residents indicated something happened during their call shift that they were not adequately prepared for and that in most of these cases, the residents did not receive information during handover that would have been useful (2). Other studies have identified pitfalls in handover as the cause of 15% of adverse

events, errors, or near misses (3). In terms of Medico-Legal risk, 43% of malpractice cases with communication breakdowns involved handovers (4).

A number of strategies for optimal handover have been suggested in the literature with standardization being noted most frequently. Other suggested strategies include providing training or education and addressing environmental issues such as lighting, interruptions and noise (4). However, there remains a lack of research evaluating the impact of formal and standardized handover processes in terms of resident training and clinical outcomes.

The PI of this project recently completed a pilot project at a major teaching hospital in Toronto that evaluated resident satisfaction with handover, training residents on adequate handover, written handover using a computerized system and clinical outcomes. The study was underpowered to show a significant change after implementation of the program due to the short study period, but the conclusion was that longer and larger scale studies are required.

The General Internal Medicine in-patient service at Vancouver General Hospital (VGH), also known as the Clinical Teaching Unit (CTU) is undergoing major changes that involve multiple handovers. The CTU does not have a formal handover process and in the setting of multiple handovers, many concerns over patient safety have been raised. A group of Internal Medicine residents have undertaken handover as their QI project, which the PI has been recruited as a co- supervisor (project end date October 2011). This proposed project will build on the UBC resident work and pilot project conducted in Toronto.

**PROJECT DETAIL**

**Goals and objectives**

The goal of this project is to implement a multi-faceted handover process on the CTU to improve patient safety around transfers of care. The process will use literature-based approaches with a goal to contribute to the existing literature on the impact of handover strategies and whether changes in resident satisfaction and more importantly, patient outcomes can be measured and demonstrated after implementation of a handover program.

At the level of the care provider, the objective is to measure the effect of a handover process on resident satisfaction with handover and resident reported adverse events related to handover. At the level of the patient, the objective is to measure patient referrals to critical care response teams, patient transfers to the intensive care unit and inappropriate code blue calls for patients with a documented do not resuscitate status.

**Clientele**

The clientele is residents rotating through the CTU. This is a mandatory rotation for Internal Medicine residents and some off-service residents in their first to third year of residency. As the CTU is undergoing major restructuring, there has been concern regarding multiple patient handovers raised at all levels including the residents, Chief Medical Residents, Internal Medicine Program Director, CTU Director and Division Head. The PI has been recruited to co-supervise a QI project on handover being conducted by a group of residents (end date October 2011) and as a member of the CTU task force committee chaired by the CTU director that meets regularly to discuss CTU changes, including patient handover. Input from these various groups and

individuals has been incorporated into this proposal and their continued involvement will be critical.

**Methods**

The handover process will include educating trainees on principles of optimal patient handover, providing a template and mnemonic for verbal and written handover and organizing structured face-to-face handover. These strategies for handover were chosen based on what is published in the literature, local needs for handover and lessons learned from the pilot project.

A major gap in the literature on patient handover is the impact of formal handover strategies on clinical outcomes. This project will not only implement a handover process to address local needs at VGH, but also study the effects on both residents and patients. Locally, in implementing a formal structure and train-the-trainer strategy for teaching handover, the project will be sustainable beyond initial implementation. Globally, our study of the impact of handover on residents and patients will contribute to a broader understanding of patient handover.

**Methodology**

*1. Educational objectives*

The handover training session will be one hour in duration, delivered once a month to residents on the CTU service. A description of the handover process will be incorporated into the CTU orientation.

Objectives of the training session are to:

a) Understand the risks associated with inadequate handover as identified in the literature.

b) Develop an approach to written and verbal handover using the published mnemonics **ANTICipate** (Administrative data, New information, Tasks, Illness state, Code status) (5) and **SIGNOUT?** (Status, Identification, General hospital course, New events, Overall health status, Upcoming possibilities, Tasks and rationale, questions) (6).

c) Practice handover skills learned from the session.

*2. Needs assessment*

Residents have been surveyed regularly on their satisfaction with patient handover since April

2011 as part of the resident QI project. Development of the program will be based on results of these surveys as well as focus groups with residents on CTU.

*3. Design/approach*

The program is based on a review of the patient handover literature, results of the pilot project conducted by the PI and local needs at VGH. This multi-faceted handover process will include handover training for residents, a template and mnemonic for verbal and written handover and structured face-to-face handover.

The training will be given as a one-hour educational session given at the beginning of the CTU rotation as described above. It will be similar to the education session taught during the pilot project using similar educational objectives. Implementation and scheduling of the session will be through the assistance of the CTU Director and Chief Medical Resident as part of the educational component of the CTU rotation.

In terms of the template and mnemonic, there are many published in the literature; ANTICipate and SIGNOUT? as described above were used during the pilot project and were well received by the participants. These will continue to be used in this project as they are comprehensive in communicating vital handover information including anticipatory events, plans and rationales, and opportunities to ask questions during handover.

All components of the handover process will be continuously evaluated using rapid cycle quality improvement methods with input from residents, staff and administrators especially given the ongoing changes with the CTU structure and changes to patient handover.

*4. Evaluation/dissemination plan*

The program will be evaluated for its effect on both care providers and patients qualitatively and quantitatively. At the level of the care providers, we will assess satisfaction with handover and resident reported adverse events related to handover through anonymous surveys and focus groups. The survey will be similar to that used in the pilot project, which was adapted by permission from a survey used by Borowitz and colleagues in assessing Pediatric residents and handover (2).

At the level of the patient, we will assess outcomes similar to those from the pilot project. Given the lack of patient outcomes reported in the literature, the pilot project team selected those that were felt to represent or had the potential to lead to an adverse event. An important lesson learned from the pilot was that our measured outcomes were rare events and hence the importance of this study to continue to measure these outcomes over a longer period. These outcomes will include: transfers to the ICU, referrals to critical care response teams (CCRT) and inappropriate code blue calls for patients with a documented "Do Not Resuscitate Order." To further expand on the pilot project, we will review the charts and handover of patients who have the outcomes listed above to determine if inadequate handover was related to the adverse event.

Dissemination of the results of this project will be through publication and presentations at local, national and international conferences. The results can be used by other institutions and disciplines to build on their patient handover process and moreover, build on our greater understanding of handover.

**Originality/Innovation**

This program is innovative because we will study the effects of implementing a multi-faceted approach to patient handover on resident satisfaction with handover and more importantly patient outcomes, which is the largest deficiency in the understanding of handover. Furthermore, the handover process to be employed will use a number of strategies identified in the literature and implement them on a medical service currently without a handover process.

**Collaborative**

This project will require collaboration between residents, staff physicians and administrators. The initial project implementation and continuous rapid cycle quality improvement will inevitably raise issues and concerns by front line workers that will need to be addressed with other front line workers and hospital administrators. Furthermore, collaboration with senior staff and administrators will hopefully allow for dissemination of the handover process from Internal Medicine to other disciplines and specialties in the hospital.

**Available Resources**

A research assistant and statistician will not be recruited for this project until funding has been secured. The individuals below will provide advice, support and collaboration.

***Dr. Barry Kassen*** - Head, UBC Division of General Internal Medicine

Clinical Professor, UBC Division of General Internal Medicine.

***Dr. Iain McCormick*** - CTU Director, VGH

Clinical Instructor, UBC Division of General Internal Medicine. Dr. McCormick has an interest in medical education and assessment.

***Dr. Graydon Meneilly*** - Head, UBC Department of Medicine

Clinical Professor, UBC Department of Medicine.

***Dr. Rohi Pai*** - Chief Medical Resident, VGH

Dr. Pai is a third year medical resident who oversees educational and administrative aspects of the CTU including patient handover. He has completed one QI project to date and is also an instructor for the QI curriculum for the Department of Medicine.

***Dr. J Mark Roberts*** - Director of Postgraduate Education, Department of Medicine Clinical Associate Professor, UBC Division of General Internal Medicine. Dr. Roberts has supervised several QI projects over the past several years. He also participated on many QI projects as Medical Manager for the Acute Medical Unit at VGH from 2000-2011.

Lastly, the PI of this project will be attending a QI course at Intermountain Healthcare from January – April 2012. As part of this course, there is a mandatory project to be completed, which this handover project may be well suited for. Intermountain Healthcare may therefore be able to provide additional support, expertise and guidance for the project.

**Building Facilities**

None required

**Equipment/Supplies**

No specific equipment is required other than usual office supplies

**Budget**

| **Budget Line Item** | **Cost** | **Grant Request** | **In-kind** |
| --- | --- | --- | --- |
| Research Assistant  (0.5 FTE @ $25.50/hr + benefits @ 22%) | $32,354 | $32,354 |  |
| Biostatistician  (80 hrs [@$46.27](mailto:@$46.27)/hr + benefits @ 22% | $4,516 | $4,516 |  |
| Laptop computer | $2,000 | $2,000 |  |

| Statistical software | $2,500 | $2,500 |  |
| --- | --- | --- | --- |
| Printing and photocopying | $1,000 | $100 |  |
| Incentive for physician survey  $5/resident x 35 residents/month x 12 months | $2,100 | $2,100 |  |
| Attendance at conference/meetings | $2,500 | $2,500 |  |
| Publication costs | $1,000 | $1,000 |  |
| Medical residents/post-graduate students |  |  | In-kind |
| Staff time |  |  | In-kind |
| **Total grant request** | **$47,970** |  | |

**Other Sources of Funding and Amounts**

None

**DISSEMINATION PLAN**

Dissemination will be through presentations at local, national and international conferences and submitted for publication in peer-reviewed journals under the responsibility of the Principle Investigator. Dissemination will be within one year of completion of the project.

**EVALUATION PLAN**

Success of the project will be two-fold. One is whether after implementation of the handover program there is a measurable change in resident and patient outcomes, the other is whether the handover program is sustainable. Evaluation of the first is dependent on rigorous evaluation using support from this grant. Evaluation of the second will be longitudinal and achieved through regular CTU end-of-rotation feedback sessions already in place.

**RISK ASSESSMENT**

| Risk | Probability | Impact | Owner | Mitigation strategy |
| --- | --- | --- | --- | --- |
| Failure to sustain written handover | Medium | High | PI, CTU director, Chief medical resident | Root cause analysis to determine causes and changes made to address these. This may require changes to the written handover format and input from other institutions with written handover in place. |
| Failure to sustain verbal handover | Medium | High | PI, CTU director, Chief medical resident | Root cause analysis to determine causes and changes made to address these. |
| Failure to sustain face- to-face handover | Medium | High | PI, CTU director, Chief medical resident | Root cause analysis to determine causes and changes made to address these. The time  and place for handover may need to be reviewed. |
| Failure to implement regular education/training sessions | Low | High | PI, Chief medical resident | A “train-the-trainer” approach to the education session will be used. Trainers and trainees may need to be revised if not successful. |
| Collection of patient outcome data – too few data points or not enough information available | Low | High | PI, research assistant | Revision of the outcome measures will be made with advice from collaborators and by performing a literature search for new studies. |

**Literature References**

1. The Joint Commission. 2009 Hospital National Patient Safety Goals. http:www.jointcommission.org/PatientSafety/Solutions/

2. Borowitz SM, Waggoner-Fountain LA, Bass EJ, Sledd RM. Adequacy of information

transferred at resident sign-out (in-hospital handover of care): a prospective survey. Qual Saf

Health Care 2008;17:6-10.

3. Jagsi R, Kitch BT, Weinstein DF, Campbell EG, Hutter M, Weissman JS. Residents report on adverse events and their causes. Arch Intern Med 2005;165:2607–2613.

4. Riesenberg LA, Leitzsch J, Massucci JL, Jaeger J, Rosenfeld JC, Patow C, et al. Residents’

and attending physicians’ handoffs: a systematic review of the literature. Acad Med

2009;84:1775-1787.

5. Vidyarthi AR et al. Managing discontinuity in academic medical centers: Strategies for a safe

and effective resident sign-out. J Hosp Med 2006;1:257-266.

6. Horwitz LI, Moin T, Green ML. Development and implementation of an oral sign-out skills

curriculum. J Gen Intern Med 2007;22:1470-1474.
